# Development and characterization of 20 polymorphic microsatellite markers for *Epinephelus marginatus* (Lowe, 1834) (Perciformes: Epinephelidae) using 454 pyrosequencing

**DOI:** 10.1590/1678-4685-GMB-2018-0067

**Published:** 2019-02-14

**Authors:** Jussara Oliveira Vaini, Kenneth Gabriel Mota, Alejandra Paola Ojeda, João Pedro Barreiros, Renata Guimarães Moreira, Alexandre Wagner Silva Hilsdorf

**Affiliations:** 1 Universidade de Mogi das Cruzes Universidade de Mogi das Cruzes Núcleo Integrado de Biotecnologia Mogi das CruzesSP Brazil Núcleo Integrado de Biotecnologia, Universidade de Mogi das Cruzes, Mogi das Cruzes, SP, Brazil; 2 Universidade dos Açores Universidade dos Açores Centro de Ecologia Evolução e Alterações Ambientais Angra do Heroísmo Portugal Centro de Ecologia, Evolução e Alterações Ambientais, Universidade dos Açores, Angra do Heroísmo, Portugal; 3 Universidade de São Paulo Universidade de São Paulo Instituto de Biociências São PauloSP Brazil Instituto de Biociências, Universidade de São Paulo, São Paulo, SP, Brazil

**Keywords:** Short tandem repeat, population genetics, conservation, fishery, marine resources

## Abstract

The dusky grouper, *Epinephelus marginatus*, is a well-known and widespread marine fish assessed as endangered by the International Union for the Conservation of Nature. Analyzing the genetic diversity of this species is, therefore, of utmost importance and necessary for conservation purposes. Microsatellites are molecular tools with advantages that are ideal for population analyses. This study provides the first set of species-specific microsatellite loci for *E. marginatus* that can be applied when assessing both intra- and interpopulation genetic variation. Twenty microsatellite loci were isolated and characterized for the dusky grouper by genotyping 20 individuals obtained from the North Eastern Atlantic Ocean (n = 4) and from the South Western Atlantic Ocean (n = 16). The number of alleles per locus varied from 2 to 11, while the observed and expected heterozygosities ranged from 0.25 to 0.94 and 0.34 to 0.89, respectively. The polymorphic information content varied from moderately to highly informative. This suite of markers provides the first specific nuclear tools for *E. marginatus* and, thus, allows to assess with more specificity different populations’ structures.

Epinephelidae, known as groupers, are considered commercially important marine resources by commercial and recreational coastal fisheries (Begossi and Silvano, 2008; Schunter *et al.*, 2011). In 2009, approximately 275,000 metric tons of the global catch were groupers (Epinephelidae), which represented approximately 90 million fish (Sadovy de Mitcheson *et al.*, 2013). Groupers play an important role in trophic foodwebs in coral and rocky reef ecosystems (Condini *et al.*, 2015). They are protogynous hermaphrodites characterized by high site fidelity, slow growth, delayed sexual maturation in males, and large body size (Marino *et al.*, 2001; Koeck *et al.*, 2014). This group of Perciformes is especially susceptible to overfishing (Morris *et al.*, 2000).

The dusky grouper, *Epinephelus marginatus* (Lowe, 1834), is broadly distributed in the Atlantic Ocean from the Mediterranean Sea to southern Africa, in the Indian Ocean northwards to Madagascar, and from the British Isles to South Africa, including the Macaronesian Archipelagos of Azores, Madeira, Canaries, and Cape Verde (Heemstra, 1991; Heemstra and Randall, 1993). On the Atlantic coast of South America, this species occurs from Rio de Janeiro (Brazil) to Patagonia (Argentina) (Rico and Acha, 2003; Irigoyen *et al.*, 2005). Fishing data have shown a 50% decline in the overall dusky grouper catches from European countries between 1994 (7699 metric tons) and 2011 (869 metric tons) (Harmelin-Vivien and Craig, 2015). This population reduction combined with their life-history has led to an assessment of Endangered A2d status by the International Union for the Conservation of Nature (Cornish and Harmelin-Vivien, 2004; Harmelin-Vivien and Craig, 2015).

Knowledge of intra- and intergenic diversity is important for planning the long-term conservation and recovery of marine fish resources through legal environmental protection and ecosystem-based fisheries management (Zhou *et al.*, 2010). Therefore, molecular markers are needed to acquire knowledge on the genetic diversity of a given species, and microsatellite markers are the most frequently used tool in marine fish (Cuéllar-Pinzón *et al.*, 2016). Although, species-specific microsatellite markers have been developed for different species of the genus *Epinephelus*: *E. quernus* – 9 loci (Rivera *et al.*, 2003); *E. septemfasciatus* – 12 loci (Zhao *et al.*, 2009a) and 22 loci (An *et al.*, 2014); *E. awoara* – 12 loci (Zhao *et al.*, 2009b); *E. fuscoguttatus* – 10 loci (Mokhtar *et al.*, 2011); *E. merra* – 13 loci (Muths and Bourjea, 2011); *E. akaara* – 12 loci (Watanabe *et al.*, 2011) and 10 loci (Xie *et al.*, 2015); *E. lanceolatus* – 32 loci (Yang *et al.*, 2011) and 24 loci (Kim *et al.*, 2016); *E. striatus* – 15 loci (Bernard *et al.*, 2012); *E. bruneus* – 28 loci (Kang *et al.*, 2013); *E. polyphekadion* – 12 loci (Ma *et al.*, 2013); *E. itajara* and *E. quinquefasciatus*, 29 and 25 loci, respectively (Seyoum *et al.*, 2013); and *E. ongus* – 18 loci (Nanami *et al.*, 2017). As species-specific microsatellite marker have not yet been developed for *E. marginatus*, previous studies of population genetic diversity used microsatellites by cross-amplification (Sola *et al.*, 1999; De Innocentiis *et al.*, 2001; De Innocentiis *et al.*, 2008; Schunter *et al.*, 2011; Elglid *et al.*, 2015; Buchholz-Sørensen and Vella, 2016; Reid *et al.*, 2016).

Now that next generation sequencing (NGS) has become more accessible, the development of species-specific microsatellite loci is much faster and less labor intensive (Kumar and Kocour, 2017). In addition, microsatellites are generally found in non-coding regions, where the substitution rate is higher than in the coding regions, and hence, the flanking regions of the microsatellites, where the primers are designed, are prone to mutations (Primmer *et al.*, 2005). Mutations in these regions may result in null alleles that affect the cross-amplification success rate (Maduna *et al.*, 2014). Another issue regarding microsatellite cross-amplification is that the ascertainment bias phenomenon may be operating, that is, the chance of the median allele length of microsatellites being longer in one species in which it was first developed than in other cognate species (Crawford *et al.*, 1998; Barbará *et al.*, 2007; Li and Kimmel, 2013). With this in mind we have now used NGS to develop a novel set of 20 specie-specific microsatellite markers to provide support to conservation management programs for the dusky grouper.

Genomic DNA was extracted from the caudal fins of five *Epinephelus marginatus* specimens using QIAGEN DNeasy blood and tissue extraction kits (QIAGEN Inc., Valencia, CA) following the manufacturer protocols, and these samples were forwarded to GenoScreen (Lille, France) for commercial microsatellite library production. The extracted DNA was fragmented (~1500 bp) by sonication (S220 Focused-ultrasonicator; Covaris, Newtown, CT, USA) and used to construct a shotgun library (GS-FLX Titanium kit; Roche Diagnostics Corporation, Branford, CT, USA), which was then sequenced in a 454 GS-FLX Titanium pyrosequencer (Roche Diagnostics Corporation). The following DNA probes were used to perform the enrichment: TG, CT, AAC, AAG, AGG, ACG, ACAT, and ACTC. The software QDD (Meglécz *et al.*, 2010) was used to identify the microsatellites from the raw sequences. A total of 5526 sequences were recovered with microsatellite motifs. Of these, 165 sequences presented simple and perfect repetitions with a minimum of five repeat motifs, where 125 resulted in dinucleotides, 26 in tri-, 13 in tetranucleotides and one pentanucleotide. Forty-eight microsatellite loci were initially selected for polymorphism assessment, and 20 of these loci amplified reliably and showed evidence of polymorphism (Table 1). Primer sets were designed from the microsatellite flanking regions using the QDD software. Twenty individuals of *E. marginatus* were analyzed to validate the panel of species-specific microsatellite markers, with 16 individuals being from the Southwestern Atlantic Ocean near Brazil (São Paulo, n = 4; Rio de Janeiro, n = 4; Parana, n = 4 and Santa Catarina coast, n = 4) and 4 individuals from the Northeastern Atlantic Ocean (Spain / Mallorca (n = 2), Greece / Cyclades Islands (n = 1) and Azores Archipelago (n = 1)).

**Table 1:** Genetic characteristic of 20 microsatellite loci in *Epinephelus marginatus*.

Locus	GenBank accession	Primer sequence (5-3)	Repeat motif	Ta (°C)	N	A	Size range (bp)	Ho	He
Ema01	KU762341	F: ACAGCAAACCATGTGAGCAG	(AC)_14_	60	20	09	158-180	0.70	0.82
		R: TGGAGTGATAGTCCTCTGTTGG							
Ema02	KU762342	F: CAGACGTATGCACTGGACCT	(TG)_7_	62	20	07	123-165	0.85	0.78
		R: ATATGTCAGCCTCCACCTCC							
Ema03	KU762343	F: CCACCATGCCCTCCAATA	(ATCT)_15_	58	19	11	125-165	0.89	0.89
		R: GAGCCAGGTGAATGACCACT							
Ema04	KU762344	F: CTGATGGAATCCACAAATTTAATC	(GATG)_9_	52	20	09	183-223	0.80	0.87
		R: TCTGACTGACAGATAACAAGAGAA							
Ema05	KU762345	F: TGCTCAGGGAGACTGACAGA	(GA)_7_	56	18	09	177-195	0.83	0.81
		R: GATGAGCAAAGAGGGCAGAG							
Ema06	KU762346	F: TTGTACGTTTGCTAATGCTGTG	(CAA)_5_	55	20	05	204-216	0.75	0.70
		R: CTGAACTGTACTCATGAACCTGC							
Ema07	KU762347	F: CCTCTACTGCTATCATGACTTCTCC	(TAC)_5_	52	12	02	205-208	0.25	0.34
		R: ACAGTTGAAATAGTGGTGCAGA							
Ema12	KU762348	F: AAAGTGCACTGTAGCCAACG	(TCC)_7_	58	17	03	221-230	0.94	0.56
		R: TGATGTGGACAACGGAAAGA							
Ema17	KU762350	F: GGGCAGTGACGGTAGACATT	(CTAT)_13_	56	20	09	142-182	0.80	0.82
		R: CGAAGACGCAATGAAACTGA							
Ema18	KU762351	F: GGACAAGTGGACATTTTGGC	(CTAT)_16_	60	20	09	175-207	0.80	0.89
		R: AACCAGGAGCTTATGTGGCT							
Ema20	KU762352	F: TGATTATGAATGCAAGAAGTGATG	(CATC)_7_	60	18	04	150-162	0.78	0.63
		R: AGGGCCGATGATCAAATGT							
Ema22	KU762353	F: GTTTGCAGTGTTGCAGTGCT	(TATG)_7_	58	20	07	111-135	0.85	0.81
		R: TAGGGTGGGATTTCAGATGC							
Ema23	KU762354	F: AACATGATCCGAATAGGCTGA	(ACAG)_7_	60	19	07	214-234	0.84	0.74
		R: CGAAGGCTCCAGGTCAGTAT							
Ema26	KU762355	F: CAGGTGGAGTGATTTCAGCA	(TTC)_8_	52	20	06	128-143	0.65	0.76
		R: TTCACCCATGGGAAGTATGA							
Ema35	MG640563	F: ACTCCCACTCTGCCTCTCAG	(AC)_14_	56	20	11	169-197	0.55	0.84
		R: ACGTGCAAATTTCTTGGACA							
Ema38	MG640564	F: TGTCTGTGACGAACTCTGCC	(TG)_11_	60	20	07	160-172	0.35	0.82
		R: CCCATCTACTGCTGGTGTCTC							
Ema42	MG640565	F: AATATGACTGATAATTTGACCACCA	(CTG)_7_	54	20	06	147-162	0.55	0.66
		R: CACCCCTAGACCAGCACAAT							
Ema43	MG640566	F: TGGGAGAAATGTGTCCTCAG	(GT)_9_	58	15	04	189-195	0.33	0.71
		R: CTGCTGCATGTTCTAGCCAA							
Ema45	MG640567	F: GGAGTTGCTAGAACCAAGCC	(TGT)_6_	56	17	04	158-167	0.47	0.62
		R: CAGCAGTCACAGAAACACGC							
Ema48	MG640568	F: TCCAAGTTACCACCTAGCCTTC	(ATCC)_5_	50	19	04	113-125	0.68	0.57
		R: ATGGATAGATGATAGATGGATGC							

Ta: Annealing temperature; N: number of individuals; A: number of alleles; Allele size in base pairs; Ho: observed heterozygosity; He: expected heterozygosity; PIC: polymorphic information content; HWE: Hardy-Weinberg Equilibrium *p-*values.

*p < 0.05 significant departure from Hardy-Weinberg Equilibrium.

Primer 5’ end labeled with M13 tail (5’TGTAAAACGACGGCCAGT 3’) (Schuelke, 2000)

For each primer set, the forward primer was 5’-end-tailed with the M13 universal sequence (5’-TGTAAAACGACGGCCAGT-3; Schuelke, 2000). The polymerase chain reactions (PCR) were performed in 20 μL reaction volumes (~100 ng genomic DNA, 1X PCR buffer, 0.25 mM dNTPs, 1.5-3.0 mM MgCl_2_, 0.5 U *Taq* DNA polymerase, 0.2 μM of the IRDye700 fluorescently labeled universal M13 primer, 10 μM of the forward primer, and 10 μM of the reverse primer) in a Veriti thermal cycler (Applied Biosystems). The amplification thermal profile for all markers had an initial denaturation at 94 °C for 5 min, followed by 35 cycles of 40 s at 94 °C, 1 min at the primer set annealing temperature (Table 1), and 40 s at 72 °C, with a final extension of 10 min at 72 °C. Amplicons were separated on 6.0% polyacrylamide gels using an automated DNA sequencer (DNA Analyzer 4300; LI-COR Biosciences, Lincoln, NE, USA). Alleles were sized using a 50-350 bp standard (LI-COR Biosciences), and genotypes were scored using SAGA v.3.3 software (LI-COR Biosciences).

The number of alleles per locus, estimates of observed (Ho) and expected heterozygosity (He), and deviations from Hardy-Weinberg expectations were determined with an exact test using the Markov chain-randomization approach (Guo and Thompson 1992) with HW-Quickcheck (Kalinowski, 2006). Linkage disequilibrium was assessed using GENEPOP v.4.2 (Rousset, 2008), and Micro-Checker v.2.2.3 (Van Oosterhout *et al.*, 2004) was used to test for null alleles and allele dropout. The polymorphic information content (PIC) was analyzed using Cervus v.3.0.7 (Kalinowski *et al.*, 2007).

Table 1 summarizes the information for each primer sequence, providing GenBank accession number, repeat motif, number of alleles, diversity estimates (Ho and He), PIC, and probability of Hardy-Weinberg equilibrium (HWE).

Among the 20 individuals, the number of alleles per locus ranged from 2 (Ema07) to 11 (Ema03 and Ema35), with an average of 6.65, while the observed and expected heterozygosities ranged from 0.25 (Ema07) to 0.94 (Ema12) and 0.34 (Ema07) to 0.89 (Ema03 and Ema18), respectively (Table 1). Sixteen loci (Ema01, Ema02, Ema03, Ema04, Ema05, Ema06, Ema07, Ema17, Ema18, Ema20, Ema22, Ema23, Ema26, Ema42, Ema45, and Ema48) were in HWE (*p* > 0.05), while four loci significantly deviated (*p* < 0.05; Ema12, Ema35, Ema38, and Ema43). Three loci (Ema35, Ema38, and Ema43) showed evidence of null alleles, but no allele dropout was detected. No statistical evidence for linkage disequilibrium was found between any of the 20 loci pairwise comparisons. The number of alleles was high and exhibited moderate to high levels of polymorphism (PIC), ranging from 0.27 (Ema07) to 0.85 (Ema02 and Ema18), with an average of 0.67. When separated in di- (0.74), tri- (0.54) and tetranucleotides (0.72), the loci that presented the highest levels of polymorphism (PIC) were the dinucleotides.

These novel polymorphic microsatellite loci developed using NGS technology will aid in achieving better resolution when assessing stock structure and population connectivity for the dusky grouper’s long-term conservation and the sustainable use of this valuable marine resource.
